# Eye-related Emergency Department Visits with Ophthalmology Consultation in Taiwan: Visual Acuity as an Indicator of Ocular Emergency

**DOI:** 10.1038/s41598-020-57804-2

**Published:** 2020-01-22

**Authors:** Eugene Yu-Chuan Kang, Wei-Chen Tai, Jui-Yen Lin, Chi-Jen Huang, Po-Han Yeh, Wei-Chi Wu, Feng-lin Wang, Laura Liu, Chi-Chun Lai, Kuan-Jen Chen

**Affiliations:** 1 Department of Ophthalmology, Chang Gung Memorial Hospital, Linkou Medical Center, Taoyuan, Taiwan; 2 College of Medicine, Chang Gung University, Taoyuan, Taiwan; 3 Department of Emergency Medicine, Chang Gung Memorial Hospital, Linkou Medical Center, Taoyuan, Taiwan

**Keywords:** Physical examination, Epidemiology, Preclinical research

## Abstract

To investigate the epidemiology of eye-related emergency department (ED) visits and to determine if visual acuity (VA) could be an indicator for determining the timing for managing ocular emergencies, we have conducted the retrospective study which included patients visited the ED for eye-related reasons and had received ophthalmology consultations at a referral center in Taiwan in 2015. Among 46,514 consultations, 5,493 were ophthalmology consultations (11.8%). After exclusion, 5,422 were eligible for analysis. Among them, 1,165 (21.5%) had not likely emergent diagnoses, 4,048 (74.7%) had likely emergent diagnoses, and 209 patients (3.9%) could not be determined. The logMAR VA was 0.31 ± 0.48, 0.66 ± 0.78, and 1.00 ± 0.94 in groups with not likely emergent, likely emergent, and undetermined diagnoses, respectively. Among all eye-related ED visits, 10.3% of patients received ophthalmologic intervention or were admitted to the ophthalmology ward. A LogMAR VA score of 0.45 (decimal equivalent of 0.4) had the highest discrimination power for identifying whether a patient needed ophthalmology intervention or admission to ophthalmology ward (area under the curve: 0.802, sensitivity: 0.800, specificity: 0.672). In our study, we found VA could be an indicator for determining the priority and time of ocular emergencies requiring ophthalmic intervention in patients visiting the ED for eye-related reasons.

## Introduction

People often visit the emergency department (ED) first for some ocular diseases. The most commonly reported eye condition in the ED is conjunctivitis followed by corneal injury, eye pain, and hordeolum^1^. Although the ED can offer rapid management of ocular problems, an excessive number of nonemergent eye-related visits may overcrowd the ED, and limit the resources available for real emergent ophthalmic and other medical issues^2^. A US study reported that approximately half of all ocular ED visits were not related to emergent conditions^3^. Informing patients on how to appropriately seek eye care was suggested^4^.

The cost of ED visits in Taiwan is covered by Taiwan’s National Health Insurance, making Taiwan health care renowned for its low cost and overloaded services^5^. The average treatment-associated expenditure and drug-associated expenditure for a single ED visit are NT$1,155 (approximately US$35.0) and NT$190 (approximately US$5.8), respectively^5^. Patients only need to pay the registration fee (from NT$200 to NT$400 [approximately US$6 to13] at tertiary medical centers) and copayment (NT$450 [approximately US$15]) for an ED visit in Taiwan^6^. Regarding to the United States, where overall, eye-related ED visits cost approximately US$2 billion annually, the mean charges of an emergent and a nonemergent eye-related ED visit are US$1,266 and US$613, respectively^3^. This considerable difference in medical fees makes visiting the ED relatively common in Taiwan.

Increased ED visits have been reported in Taiwan in recent years^5^. The Chang Gung Memorial Hospital (CGMH) Linkou Medical Center, a tertiary referral medical center in northern Taiwan, has had the most ED visits for years in Taiwan. There were 215,400 ED visits at the CGMH Linkou Medical Center, among 7.2 million ED visits nationwide in 2015^7^. In addition, approximately 450 ED ophthalmology consultations were requested every month by ED physicians at the CGMH Linkou Medical Center.

In addition to the overloaded service, the Taiwan triage and acuity scale, developed for general ED visits, was deemed to not be appropriate for determining ocular emergencies in Taiwan^8^. Hence, eye-related ED services became congested and inefficient in Taiwan. Therefore, we conducted this study not only to understand the epidemiology of all-cause eye-related ED visits with ophthalmology consultation in Taiwan but also to investigate visual acuity (VA) as an indicator for determining the timing for managing ocular emergencies.

## Material and Methods

### Study participants

This retrospective cohort study was approved by the Institutional Review Board of Chang Gung Memorial Hospital, Taoyuan, Taiwan (No. 201600080B0). The requirement of written informed consent was waived by the board. All methods were performed in accordance with the relevant guidelines and regulations. All data were reviewed anonymously in adherence with the principles of the Declaration of Helsinki. The medical records from ED ophthalmic consultations between January 1, 2015 and December 31, 2015 were reviewed. A medical record was excluded if the patient was discharged against medical advice or if the record was not obtainable from the electronic system.

### Data collection

Based on the review of medical records, clinical information was collected including demographics, visiting time, consultation time, chief complaint, trauma events, VA, diagnosis, outcomes, ED revisiting, and outpatient clinic visits after discharge from the ED. The visiting time was divided in three working shifts as follows: morning shift (8 am to 4 pm), evening shift (4 pm to 12 am), and midnight shift (12 am to 8 am). A traumatic event was defined as a clearly-stated eye injury according to a patient’s chief complaints. VA was measured at the ED through any available method (with prepared glasses, using a Snellen near letter chart, or using a Snellen E (Landolt C or other Snellen [not E]) chart at 6 m), and the result was converted to a logMAR VA as described in a previous publication^9^. We used a logMAR VA of 2 to represent the counting fingers vision test and logMAR VA of 2.3, 2.8, and 3 to represent hand movement, light perception, and no light perception, respectively^10^. The VA from the eye with the worse value was used if bilateral eyes were involved. Outcomes included discharge after treatment, admission, and surgical intervention during the visit. Primary diagnosis was made by ophthalmologists after consultations. Outpatient clinic follow-up was identified if the patient had been followed at our clinic after being discharged from the ED or the ward.

### Categorization

Patients were classified into three groups, namely the not likely emergent, likely emergent, and undetermined groups, according to the diagnosis and in accordance with the classification in the study of Channa *et al*.^3^. The classification depended on whether the diagnosis was likely to involve an ocular emergency, not likely to involve an ocular emergency, or involved an undetermined emergency. Surgery-related problems included surgical complications, postoperative follow-up, and disorders directly related to surgery. Nonocular diagnoses included head injury without ocular involvement, hypertension, and child abuse. Other emergencies included nonspecific disorders without definite diagnosis.

### Statistics

Categorical variables were presented as numbers and percentages, whereas continuous variables were described as mean ± standard deviation. For the descriptive analysis, the chi-square test, analysis of variance, and independent t test were used for categorical and continuous variables. A receiver operating characteristic (ROC) curve was created, and the area under the curve (AUC) was calculated. Youden’s index was used to summarize the measurements of the ROC curve and capture the best performance of the test. Statistical significance was set at *P* < 0.05. Statistical analysis was performed using IBM SPSS Statistics version 23 (SPSS Inc., Chicago, IL, USA).

## Results

**Figure 1:**
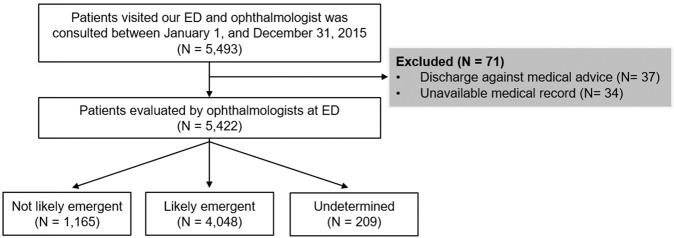
Flow chart of the inclusion and exclusion of participants. ED: emergency department.

From January 1, 2015 to December 31, 2015, approximately 215,400 people visited the ED at the CGMH Linkou Medical Center, and 46,514 of them received a specialty consultation. The number of consultations for various specialists is presented in Supplementary Table 1. There were 5,493 ophthalmology consultations (2.6% of all visits and 11.8% of all consultations). We excluded 71 patients that were discharged against medical advice (n = 37) or whose medical records were unavailable (n = 34). A total of 5,422 patients were included, and 1,165 (21.5%), 4,048 (74.7%), and 209 (3.9%) of them were classified into the not likely emergent, likely emergent, and undetermined groups, respectively (Fig. 1).

The frequency of ophthalmic diseases in each group is listed in Table 1. In the not likely emergent group, conjunctivitis (9.9% of all patients), photokeratitis (2.5%), and infection of the skin (2.4%) were the primary diagnoses. In the likely emergent group, ocular surface injury (26.4%) was the most frequent diagnosis, followed by burn injury (10.3%) and retinal detachment (6.1%). Surgery-related problems (2.1%) were the main diagnoses in the undetermined group.

**Table 1:** Distribution of the diagnoses classified as not likely emergent, likely emergent, and undetermined.

Diagnosis	Number	%
**Total**	**5,422**	**100**
**Not likely emergent**	**1,165**	**21.5**
Conjunctivitis	535	9.9
Photokeratitis	135	2.5
Infection of skin^a^	129	2.4
Subconjunctival hemorrhage	111	2.1
Vitreoretinal degeneration	109	2.0
Unspecific headache	47	0.9
Retinopathy^b^	38	0.7
Inflammation of skin^c^	23	0.4
Cataract	16	0.3
Dislocation of lens	7	0.1
Myopia	7	0.1
Dry eye	5	0.1
Nasolacrimal duct stenosis	3	0.1
**Likely emergent**	**4,048**	**74.7**
Ocular surface injury	1,429	26.4
Burn injury	557	10.3
Retinal detachment	328	6.1
Contusion of eyeball	318	5.9
Ocular hypertension	248	4.6
Facial bone fracture	209	3.9
Corneal ulcer	142	2.6
Hyphema	138	2.6
Vitreoretinal hemorrhage	118	2.2
Open globe injury	114	2.1
Herpes infection	110	2.0
Laceration of periocular skin	98	1.8
Uveitis	83	1.5
Endophthalmitis^d^	45	0.8
Neuropathy^e^	42	0.8
Retinal vascular occlusion	32	0.6
Orbital infection	20	0.4
Retinal breaks	14	0.3
Orbital inflammation	3	0.1
**Undetermined**	**209**	**3.9**
Surgery-related problems^f^	112	2.1
Non-ocular diseases	21	0.4
Cerebrovascular accident	7	0.1
Other	69	1.3

^a^Including hordeolum and cellulitis outside the orbit.

^b^Including diabetic retinopathy, hypertensive retinopathy, and myopic retinopathy.

^c^Including chalazion and dermatitis.

^d^Including postoperative and endogenous endophthalmitis.

^e^Including cranial nerve palsy and peripheral neuropathy.

^f^Including complications during cataract surgery, postoperative wound check, unspecific discomfort after ocular surgery, and nasolacrimal stent dislocation.

The results of the comparison between each group were analyzed and are presented in Table 2. Male patients were predominant (61.3%) in the likely emergent group (*P* < 0.001). The likely emergent group also had the longest consultation time (approximately 88.8 minutes), the most trauma events (57.8%), and the shortest chief complaint duration (79.7% within 1 day) (all *P* < 0.001). Patients with eye-related complaints visited our ED most frequently during the evening shift (43.9%), less frequently during the morning shift (34.9%), and least frequently during the midnight shift (21.2%). Visits during the midnight shift were more frequent in the not likely emergent group (27.1%) than in the other groups (*P* < 0.001). The overall referral acceptance rate was 16.8%, and this rate was highest in the undetermined group (22.7%) (*P* < 0.001), which included patients referred from other clinics for postoperative complications. Most patients in the not likely emergent group were discharged from the ED after evaluation and treatment (94.6%), but this group had the lowest rate of clinic follow-up (38.4%) (all *P* < 0.001). Regarding the outcomes of ED visits, none of the patients in the not likely emergent group received surgical intervention or were admitted to the ophthalmology ward. However, 12.7% and 12.9% of patients underwent a surgical intervention and 11.1% and 10.5% of patients were admitted to the ophthalmology ward, respectively, in the likely emergent group and undetermined diagnosis group. In total, 7.1% of patients were admitted to other wards after consultation (5.4% in the not likely emergent group, 7.3% in the likely emergent group, and 12.4% in the undetermined group), and 54.0% of patients returned to the ophthalmology clinic for follow-up (38.4% in the not likely emergent group, 57.8% in the likely emergent group, and 66.5% in the undetermined group). In addition, only 1% of all patients revisited our ED within 72 hours after their discharge from the ED. The logMAR VA was worst in the undetermined group (1.00; decimal equivalent 0.1 or the equivalent VA in Snellen units of 20/200) followed by the likely emergent group (0.66; decimal equivalent of approximately 0.2) and the not likely emergent group (0.31; decimal equivalent of approximately 0.5).

**Table 2:** Patient characteristics between the not likely emergent, likely emergent, and undetermined diagnosis groups.

Factor	Total	Not likely emergent	Likely emergent	Undetermined	*P* value
	N = 5,422	N = 1,165	N = 4,048	N = 209	
Male (%)	3,212 (59.2)	617 (53.0)	2,483 (61.3)	112 (53.6)	**<0.001**
Age (years)^a^	42.3 ± 20.0	38.8 ± 21.9	42.8 ± 19.3	52.1 ± 19.9	**<0.001**
Time of ED visit					
Morning shift (%)	1,893 (34.9)	366 (31.4)	1,430 (35.3)	97 (46.4)	**<0.001**
Evening shift (%)	2,382 (43.9)	483 (41.5)	1,816 (44.9)	83 (39.7)	
Midnight shift (%)	1,147 (21.2)	316 (27.1)	802 (19.9)	29 (13.9)	
Referral from other institutes (%)	911 (16.8)	142 (12.2)	722 (17.8)	47 (22.5)	**<0.001**
Consultation time (mins)^a^	84.8 ± 62.0	70.1 ± 49.9	88.8 ± 64.7	88.6 ± 57.1	**<0.001**
Trauma (%)	2,551 (47.0)	200 (17.2)	2,341 (57.8)	10 (4.8)	**<0.001**
Chief complaint duration					
<1 day (%)	4,182 (77.1)	817 (70.1)	3,225 (79.7)	140 (67.0)	**<0.001**
1–7 days (%)	861 (15.9)	242 (20.8)	570 (14.1)	49 (23.4)	
>7 days (%)	379 (7.0)	106 (9.1)	253 (6.3)	20 (9.6)	
Outcome					
Discharge after treatment (%)	4,563 (84.2)	1,102 (94.6)	3,300 (81.5)	161 (77.0)	**<0.001**
Oph interventions or admission (%)	558 (10.3)	0	529 (13.1)	29 (13.9)	**<0.001**
Oph interventions (%)	541 (10.0)	0	514 (12.7)	27 (12.9)	**<0.001**
Oph admission (%)	473 (8.7)	0	451 (11.1)	22 (10.5)	**<0.001**
Admission to other wards (%)	386 (7.1)	63 (5.4)	297 (7.3)	26 (12.4)	**0.001**
ED revisit within 72 hours (%)	54 (1.0)	11 (0.9)	37 (0.9)	6 (2.9)	**0.021**
Out-patient clinic follow-up (%)	2,927 (54.0)	447 (38.4)	2,341 (57.8)	139 (66.5)	**<0.001**
LogMAR visual acuity^a^	0.6 ± 0.8	0.3 ± 0.5	0.7 ± 0.8	1.0 ± 0.9	**<0.001**

ED: emergency department, Oph: ophthalmology.

^a^The value is presented as mean ± standard deviation.

**Figure 2:**
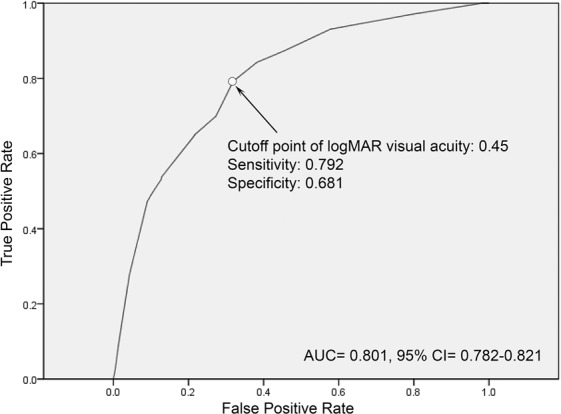
Receiver operating characteristic curve of logMAR visual acuity and cutoff analysis in the determination of the requirements for ophthalmologic intervention or admission to the ophthalmology ward. AUC: area under the curve, CI: confidence interval.

The diagnostic performance of VA as an indicator of ocular emergency is illustrated in Fig. 2. The AUC was 0.801 with a 95% confidence interval of 0.782–0.812. The cutoff point of VA at a logMAR VA of 0.45 (decimal equivalent of approximately 0.4 or the equivalent VA in Snellen units of 20/50) had the highest Youden’s index value with a sensitivity of 0.792 and a specificity of 0.681. Distinct values of sensitivity and specificity in various VA are presented in Table 3.

**Table 3:** Sensitivity and specificity for various values of logMAR visual acuity as an indicator of whether ophthalmology intervention or admission to the ophthalmology ward is required.

LogMAR VA	Decimal Equivalent	Snellen Equivalent	Sensitivity	Specificity
0.05	0.9	20/22	0.97	0.21
0.25	0.6	20/35	0.88	0.54
0.45	0.4	20/56	0.79	0.68
0.55	0.3	20/70	0.70	0.72
0.75	0.2	20/112	0.65	0.78
1.05	0.1	20/224	0.54	0.87
2.00	CF	CF	0.28	0.96
2.30	HM	HM	0.09	0.99

VA: visual acuity, CF: counting fingers, HM: hand movement.

## Discussion

This is the first study to analyze all-cause eye-related ED visits related to ophthalmology consultation in Taiwan. Ophthalmology consultations were the most common ED consultations at our hospital. Based on the results from our study, 74.7% of cases were likely emergent and 21.5% of cases were not likely emergent. Among all patients, only 10.3% received surgical intervention during their visit or were admitted to the ophthalmology ward, and only half of all patients (54%) returned to our clinic for follow-up after their ED visit. A LogMAR VA of 0.45 (decimal equivalent of approximately 0.4) exhibited the highest performance for determining whether a patient required immediate surgical intervention or ophthalmology ward admission.

According to the US studies, approximately 1.5% to 3.4% of patients visiting the ED came for ophthalmic reasons^1,4^. Regarding eye-related ED visits, 41.2% were emergent and 44.3% of were nonemergent from 2006 to 2011^3^, and 23.0% were related for nonemergent ocular conditions from 2001 to 2014 according to another study^4^. The most frequent ophthalmic disorders diagnosed were conjunctivitis (33.8%) followed by corneal injury (13.1%), which were also the most common nonemergent and emergent diagnoses, respectively^1^. Excessive unnecessary ED visits with ocular complaints have been reported^11^. To clarify the prioritization of treatment at EDs, the Taiwan triage and acuity scale was designed to determine the urgency of each ED visit and redirect nonemergent patients to an outpatient setting. However, based on the conclusions from a previous study, the Taiwan triage and acuity scale was not suitable for evaluating ocular emergencies among patients visiting the ED for eye-related reasons, and the establishment of a clinical indicator was suggested for differentiating true ocular emergencies^8^.

Compared with the aforementioned US studies, the rate of ED visits for ophthalmic reasons was similar (2.6%) in our study, whereas the rate of likely emergent ocular conditions was relatively higher (74.7%). However, only 10.3% of all patients and 13.1% of patients in the likely emergent group received ophthalmologic interventions or were admitted to the ophthalmology ward, which indicated that some of the patients with a likely emergent diagnosis did not require immediate or intensive treatment. In the likely emergent group, the most frequent diagnosis was ocular surface injury. Although ocular surface injury was the most common emergent diagnosis, only 1 of 1,429 patients received immediate intervention, and only 484 of 1,429 (33.9%) patients returned to our clinic for follow-up after being discharged from the ED. This suggested that most visits for ocular surface injury presented a low risk of ocular emergency and had a favorable prognosis with medical treatment only. In addition, some ocular surface injuries may be associated with small corneal abrasions caused by nonemergent problems, such as conjunctivitis or dry eye syndrome. Those patients can be treated using topical antibiotics and lubricant eye drops initially and referred to an outpatient clinic for further management after 24 hours^12^.

In this study, we used VA as an indicator for identifying real ophthalmic emergency, and our result suggested that VA had a good predicting power for eye-related ED visits requiring ophthalmologic intervention or admission to the ophthalmology ward (AUC = 0.801). The cutoff VA value was a logMAR score of 0.45 (decimal equivalent of approximately 0.4), which had the best performance in differentiating likely from not likely ocular emergencies. We also observed a high sensitivity of 0.97 when a patient presented with a logMAR VA of 0.05 (decimal equivalent of approximately 0.9) and a high specificity of 0.96 and 0.99 when a patient presented with a logMAR VA of 2.00 (counting fingers) and 2.30 (hand movement), respectively. Rossi *et al*. proposed an eye-dedicated triaging system, the RESCUE, using redness, pain, loss of vision, and open eye risks as parameters^13,14^. However, the RESCUE is designed for referral eye care centers exclusively dedicated to ophthalmology. Some of the parameters may not be standardized for measurement by triage nurses or doctors in general hospitals. Considering that poor vision was associated with ocular injuries requiring hospitalization^15^ and VA could be rapidly estimated^16^, we suggested using VA as a single factor to determine ocular emergency. In addition, VA could be effortlessly measured at the ED by trained triage nurses^17,18^.

Although initial VA can provide a rapid method for screening for ocular emergencies, it does not essentially exclude all severe ocular problems^19^, such as retinal break or detachment without macular involvement, which are usually presented with floaters, photopsia, or visual field defect at an early stage^20^. Therefore, the measurement of VA for eye-related ED visits could facilitate the prioritization of patient management. For example, a patient presenting at a mid-night shift with a good VA could be scheduled for review after a morning shift starts. Referral to ophthalmology outpatient settings remains essential for patients with eye-specific complaints. In addition, increased surgical risks were reported in surgeons with sleep deprivation due to the loss of surgeons’ dexterity^21^. Using VA for determining the time for managing eye-related ED visits could decrease the risk caused by sleep deprivation of a surgeon in a cost-effective fashion.

The distribution of the disease presentation in our study may not be comparable to other institutes generally. Because our hospital is one of the large-scale referral centers with 24-hour ophthalmology consultation in Taiwan, more severe ocular diseases could be seen. At the midnight shift, patients with eye-related problems could only visit referral centers with 24-hour ophthalmology consultation. This may be the reason for higher not-likely emergent eye visits presenting during the midnight shift.

This study had some limitations. First, the study only recorded ED visits with ophthalmology consultation for 1 year. A longer study duration may be required to evaluate epidemiology changes in eye-related ED visits. Second, we included patients that had received a consultation in ophthalmology clinics only. Some patients who visited the ED for eye-related problems and who did not receive an ophthalmology consultation based on ED physicians’ judgment may have caused missing information. Third, we did not unify the measurement of VA in our study because of the large variability of patient conditions resulting from the ED setting. However, our study aimed to establish a VA value for rapidly screening emergent eye diseases in an ED setting, and a simplified VA measurement was confirmed to provide crucial clinical information during ED visits^16^. This study also had some strengths. Given that all patients received a consultation in an ophthalmological setting, the ocular exams and diagnoses were ascertained by ophthalmologists. The accuracy of the diagnoses should be more robust compared with previous studies that used disease codes. The study was conducted at the medical center with the most ED visits and emergent ophthalmology consultations in Taiwan; therefore, it might offer a meaningful epidemiology of eye-related ED visits in the general population. In addition, compared with a French study that reported a total 781 eye-related ED visits at three medical centers requesting ophthalmology consultations during 3 months^22^, the number of eye-related ED visits with ophthalmology consultations in our study at a single medical center was relatively sufficient.

## Conclusion

In conclusion, this is the first study to analyze the epidemiology of eye-related ED visits with ophthalmology consultation in Taiwan. In our study, we noted that approximately one-fifth of the visits were not likely emergent. Among the overall and likely emergent visits, only 10% and 13% of patients, respectively, received immediate ophthalmologic interventions or were admitted to the ophthalmology ward. This indicated that a considerable proportion of eye-related ED visits may be not truly emergent. VA could be an indicator for determining if ophthalmologic intervention or admission to the ophthalmology ward is required and help with determining the time for managing eye-related ED visits.

## Supplementary information

Supplementary appendix.
