# Supplementary material for: Eye-related Emergency Department Visits with Ophthalmology Consultation in Taiwan: Visual Acuity as an Indicator of Ocular Emergency
Source: Sci Rep. 2020 Jan 22;10:982. doi: 10.1038/s41598-020-57804-2 (PMC6976571; doi:10.1038/s41598-020-57804-2)
Supplement: Supplementary file 1 — Supplementary appendix. [file 41598_2020_57804_MOESM1_ESM.docx]

**Supplementary Appendix**

**Eye-related Emergency Department Visits with Ophthalmology Consultation in Taiwan: Visual Acuity as an Indicator of Ocular Emergency**

Eugene Yu-Chuan Kang, MD, Wei-Chen Tai, MD, Jui-Yen Lin, MD, Chi-Jen Huang, MD, Po-Han Yeh, MD, Wei-Chi Wu, MD, PhD, Feng-lin Wang, MD, Laura Liu, MD, PhD, Chi-Chun Lai, MD, Kuan-Jen Chen, MD

| **Supplementary Table 1.** Number of consulted specialties at the emergency department. | | |
| --- | --- | --- |
| **Department** | **Number** | **%** |
| **Total** | **46,514** | **100** |
| Ophthalmology | 5,493 | 11.8 |
| Orthopedic Surgery | 4,877 | 10.5 |
| Otolaryngology | 4,334 | 9.3 |
| Obstetrics and Gynecology | 4,013 | 8.6 |
| Trauma and Emergency Surgery | 3,938 | 8.5 |
| Plastic Surgery | 3,190 | 6.9 |
| Neurosurgery | 2,942 | 6.3 |
| Nephrology | 2,188 | 4.7 |
| Cardiology | 1,996 | 4.3 |
| Cardiovascular Surgery | 1,717 | 3.7 |
| Dentistry | 1,671 | 3.6 |
| Gastroenterology | 1,355 | 2.9 |
| Urology | 1,293 | 2.8 |
| Neurology | 1,221 | 2.6 |
| General Surgery | 1,181 | 2.5 |
| Pulmonary Medicine | 1,138 | 2.4 |
| Psychiatry | 1,134 | 2.4 |
| Colon Rectal Surgery | 822 | 1.8 |
| Dermatology | 639 | 1.4 |
| Thoracic Surgery | 352 | 0.8 |
| Others^a^ | 1,020 | 2.2 |

^a^ Including therapeutic radiology, pediatric surgery, hematology,

oncology, anesthesiology, infectious disease, endocrinology,

rheumatology, rehabilitation, and general medicine.
